# Hearing preservation in cochlear implant recipients: A cross‐sectional cohort study

**DOI:** 10.1111/coa.13927

**Published:** 2022-03-15

**Authors:** Ellen Kant, Saad Jwair, Hans G.X.M. Thomeer

**Affiliations:** ^1^ Department of Otorhinolaryngology and Head & Neck Surgery University Medical Center Utrecht Utrecht the Netherlands; ^2^ University Medical Center Utrecht Brain Center University Medical Center Utrecht Utrecht the Netherlands

**Keywords:** cochlear implantation, electrode array, hearing preservation, residual hearing, speech perception

**Key points**

- Half of all adult cochlear implant recipients had residual hearing at lower frequencies prior to surgery.
- Only a minority of CI recipients retain their residual hearing after cochlear implantation.
- Lateral wall electrode arrays are preferred for hearing preservation.
- Speech perception with CI not improved in CI recipients with preserved residual hearing.
- Surgical experience had no influence on hearing preservation outcomes.

## 1 INTRODUCTION

The success of cochlear implants (CI) has led to a more diverse population of CI recipients. Originally, only patients with near‐total hearing loss were eligible for a CI. Nowadays, however, more and more CI recipients have considerable residual hearing at lower frequencies prior to implantation. This development has led to a renewed focus on achieving hearing preservation (HP) in the CI field.^1^

HP might be important for three main reasons. (1) CI recipients might benefit from their residual hearing as it can be used for electric‐acoustic stimulation (EAS).^2^ The use of EAS can improve speech perception in difficult listening situations with background noise or even improve musical melody recognition.^3^ (2) By achieving HP, a new category of patients can benefit from a CI, for example patients suffering from tinnitus.^4^ (3) Preventing hair cell loss might potentially halt auditory nerve degeneration to a degree, resulting possibly in better electric hearing outcomes in CI recipients.^5^

Although there is no lack of studies investigating HP, no consensus exists on how to achieve HP.^6^ This study aimed to provide a comprehensive retrospective overview of HP outcomes of a general CI population of a large tertiary referral centre. In addition, the effect of HP on speech perception outcomes, and other factors on HP, including surgical experience, were investigated.

## 2 METHODS

### 2.1 Patients

A retrospective cohort study was performed of adult patients who underwent cochlear implantation in a single tertiary referral centre (UMC Utrecht) from 01 January 2015 to 23 October 2020. The patients were identified using the CI registration list. Patients with a preoperative pure‐tone average threshold (PTA_low_) of the 125, 250 and 500 Hz frequencies <80 dB HL (decibels hearing level) were eligible for inclusion. The following exclusion criteria were used:

1. revision surgery
2. implantation at age <18 years
3. history of otologic surgery in the implanted ear
4. signs of acute or chronic middle ear infections and/or mastoiditis during surgery
5. incomplete electrode insertion
6. inner ear malformations or otosclerosis

All procedures performed in this study were in accordance with the ethical standards of the institutional research committee and the Declaration of Helsinki. Ethics approval for this study was obtained from the local medical ethical review board of UMC Utrecht (METC file: 21/018). Strobe reporting guideline was used for this manuscript.

All CI recipients receive at least one year after surgery rehabilitation services. In the first three months, at least 4 sessions are planned with audiologists and speech therapists. Evaluation sessions are held at 3 and 12 months postoperative.

### 2.2 Data extraction

The following data were collected from the electronic medical records: age at implantation, cause of deafness, side of implantation, date of implantation, name of surgeon, electrode‐array type, the use of perioperative corticosteroids (local or systemic), the use of hyaluronic acid, pre‐ and postoperative PTA_low_ outcomes of the implanted and contralateral ear, and consonant/vowel/consonant (CVC)‐word test outcomes.

### 2.3 Data analysis

The pure‐tone audiogram outcomes were subtracted from medical records with SAS Enterprise Guide. The HP scores of 125, 250 and 500 Hz were separately calculated by adapting the equation of Skarzynski et al. 2013:

$$\mathrm{HP}\;\left(\%\right)=\left[1‐\frac{\;\text{(}\mathrm{thresholdPost}‐\;\;\mathrm{thresholdPre})}{\left(\mathrm{outputmax}‐\mathrm{thresholdPre}\right)}\right]*100$$

HP = hearing preservation in %; thresholds in decibels hearing level (dB HL); outputmax = maximal detectable hearing level of the audiological setup at the tested frequency (i.e. 125 Hz = 70 dB HL, 250 Hz = 85 dB HL and 500 Hz = 115 dB HL).

The HP scores were categorised, also according to consensus paper of Skarzynski et al. 2013, as follows: complete HP (>75%), partial HP (>25%–75%), minimal HP (0%–25%) and complete loss of hearing (no measurable hearing). These HP scores were also checked manually. In cases with a difference between the pre‐ and postoperative hearing level at the same frequency of 5 dB, which is equal to the margin of error of the audiometry, HP on this frequency was considered as complete HP.

The CVC‐word test outcomes were extracted preoperatively (approximately 6 months prior to surgery), and postoperatively at 3 and 12 months. The preoperative CVC scores were obtained with hearing aids in both ears. Postoperative CVC scores were obtained with activated CI and hearing aid contralaterally to adequately determine the speech perception shifts. These CVC scores were obtained in a situation without background noise. Patients with one‐sided hearing impairment were included in the analyses for HP, but excluded for CVC‐score analyses.

The pure‐tone audiometry outcomes were extracted of the contralateral non‐implanted ear in 45 patients to evaluate deterioration of hearing levels irrespective of surgery. The electrode‐array type was categorised as perimodiolar or lateral wall. The mid‐scala electrode array of Advanced Bionics was classified as perimodiolar electrode array, because it is precurved.

## 3 RESULTS

**FIGURE 1:**
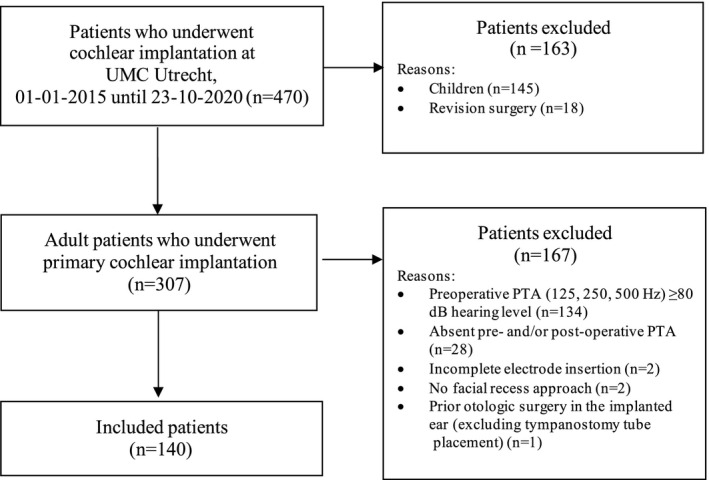
Patient selection process. PTA = pure‐tone average

A total of 470 patients underwent cochlear implant surgery. Of this group, 307 patients were adult and underwent primary cochlear implantation. In total, 140 patients were eligible for inclusion (46% of all adults with primary cochlear implantation). See Figure 1 for the in/exclusion flowchart. At time of implantation, mean age of the included patients was 61 years (SD: 17), with 64% male. Most patients suffered from bilateral idiopathic progressive sensorineural hearing loss (*n* = 101, 73%). See Table 1 for the demographics.

**TABLE 1:** Demographics of included patients

Patient demographics	N = 140 (%)
Age at implantation, *mean (SD)*	61 (17)
Gender	
Female	51 (36)
Male	89 (64)
Medical indication for cochlear implantation	
Bilateral IPSNHL	101 (72)
Sudden deafness unilateral	7 (5)
Usher syndrome	5 (4)
DFNA9 mutation	5 (4)
Other	22 (15)

Abbreviations: IPSNHL, idiopathic progressive sensorineural hearing loss; N, number; SD, standard deviation.

### 3.1 Hearing preservation

Descriptive data are shown in Table 2. Complete HP was achieved in 14 patients (10%), partial HP in 36 patients (26%), minimal HP in 42 patients (30%) and complete loss of residual hearing in 48 patients (34%). At random, PTA_low_ outcomes were extracted of the contralateral non‐implanted ear for 45 patients, showing no difference between pre‐ and postoperative outcomes (*p *> 0.05). Average time between cochlear implantation and postoperative tone audiogram was 88 days for all patients (*p *> 0.05, between groups).

**TABLE 2:** Comparison of several factors between the hearing preservation groups

		All	Complete HP	Partial HP	Minimal HP	Complete hearing loss	Statistics
Patient dependent factors; *n* (%)							
Mean age at implantation, (SD)		61 (17)	53 (17)	60 (16)	65 (16)	61 (17)	H (3) = 8.01 ***p *= 0.046^A^** *r* = −0.21^B^
Gender	F	51	3 (6)	14 (27)	12 (24)	22 (43)	χ^2^ (3) = 4.41
	M	89	11 (12)	22 (25)	30 (34)	26 (29)	*p *> 0.05^C^
Side	L	70	6 (9)	22 (31)	22 (31)	20 (29)	χ^2^ (3) = 3.49
	R	70	8 (11)	14 (20)	20 (29)	28 (40)	*p *> 0.05^C^
Bilateral IPSNHL		101	10 (10)	33 (33)	30 (30)	28 (28)	Fisher's = 20.77 *p *> 0.05^D*^
Mean preoperative PTA_low,_ dB HL, (SD)		57	53 (20)	49 (16)	60 (16)	62 (15)	*r* = −0.19^B^
Patient independent factors; n (%)							
Electrode	PM	66	4 (6)	13 (20)	18 (27)	31 (47)	χ^2^ (3) = 9.87
	LW	74	10 (14)	23 (31)	24 (32)	17 (23)	***p *= 0.019^C^**
Intravenous corticosteroid	Yes	48	9 (19)	12 (25)	15 (31)	12 (25)	χ^2^ (3) = 7.48
	No	92	5 (5)	24 (26)	27 (29)	36 (39)	*p *> 0.05^C^
Local corticosteroid	Yes	8	3 (37.5)	3 (37.5)	2 (25)	0 (0)	Fisher's = 8.75
	No	132	11 (8)	33 (25)	40 (30)	48 (26)	***p *= 0.012^D^**
Hyaluronic acid	Yes	105	11 (11)	29 (28)	32 (30)	33 (31)	χ^2^ (3) = 1.72
	No	35	3 (9)	7 (20)	10 (29)	15 (42)	*p *> 0.05^C^
Total		140	14 (10)	36 (26)	42 (30)	48 (34)	

Note

Statistical tests: A, Kruskal–Wallis test; complete vs. minimal HP, B, Pearson correlation coefficient, C, Chi‐square test for contingencies, D, Fisher's exact test.

^*^Defined for all medical indications, only bilateral idiopathic progressive sensorineural hearing loss displayed in table.

Abbreviations: dB, decibel; F, female; HL, hearing level; HP, hearing preservation; IPSNHL, idiopathic progressive sensorineural hearing loss; L, left; LW, lateral wall electrode array; M, male; n, number; PM, perimodiolar electrode array; PTA_low_, pure‐tone average of 125, 250 and 500 Hz, ; R, right; SD, standard deviation.

### 3.2 Patient dependent factors

The mean age of all patients was 61 years. This was only significantly lower when comparing complete HP group with minimal HP group (*H*‐test (3) = 8.01, *p* = 0.046). However, there was a very weak correlation between age and HP as continuous measure (*r* = −0.21). Gender (χ^2^ (3) = 4.41, *p *> 0.05) and side of implantation (χ^2^ (3) = 3.49, *p *> 0.05) were not different between HP groups. Weak correlation was observed between preoperative PTA_low_ and HP as a continuous measure (*r* = −0.19). Taken together, no baseline differences between HP groups were identified.

### 3.3 Patient independent factors

Looking at electrode array, PM arrays were used in 66 patients (47%), of which 4 had complete HP (6%) and 31 complete hearing loss (47%). A LW array was used in 74 patients. Ten patients had complete HP (14%), and 17 patients had no preservation of their hearing (23%). Patients with LW arrays had better HP than patients with PM arrays (χ^2^ (3) = 9.87, *p* = 0.019).

Forty‐eight patients (34%) received intravenous corticosteroids during surgery. Total dose ranged between 4 and 24 mg, in 1–3 administrations. The use of intravenous corticosteroids was not associated with HP (χ^2^ (3) = 7.48, *p *> 0.05). Local corticosteroids were administered in eight patients, of which three had complete HP and five had partial HP. The use of local corticosteroids seems to be associated with better HP (Fisher's = 8.75, *p *= 0.012), although all 8 patients also received a LW array. Hyaluronic acid was received by 105 patients (75%), with no differences between HP groups (χ^2^ (3) = 1.72, *p *> 0.05).

### 3.4 Surgical experience

The majority of the included implantations were done by one surgeon (*n* = 102), and these HP outcomes were analysed. Before 2015, this surgeon performed around 40 implantations. There was no correlation between experience in days and HP (all patients: *r* = −0.05, *p *> 0.05; only PM arrays: *r* = 0.19, *p *> 0.05; only LW arrays: *r* = −0.07, *p *> 0.05). The remainder of the patients (*n *= 38) was implanted by one of five surgeons, and sample sizes were too low (range 2–19) to show a meaningful distribution of HP.

### 3.5 Speech perception

**FIGURE 2:**
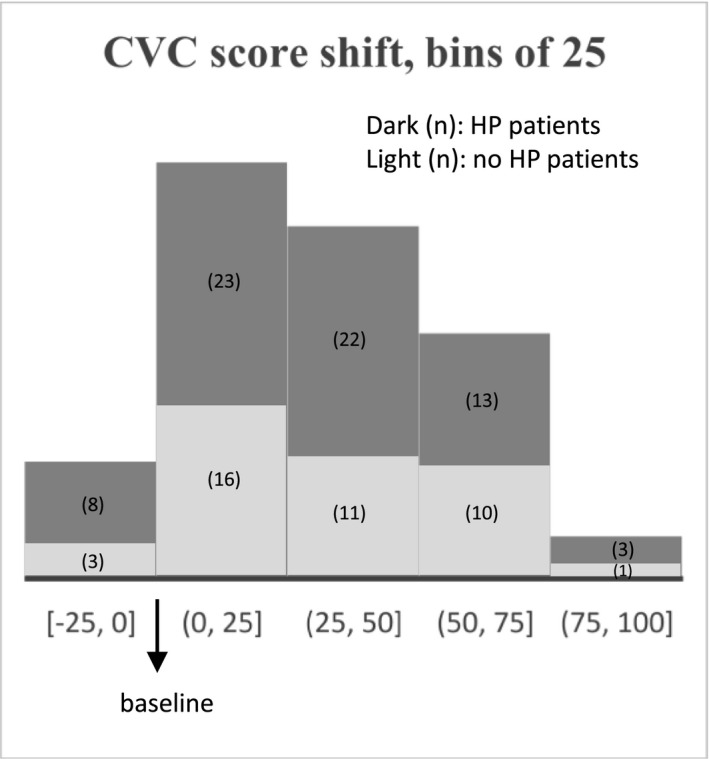
Postoperative CVC scores of patients with minimal to complete hearing preservation (HP) and patients with total loss of residual hearing (no HP)

A total of 110 CI recipients had CVC scores available at 3 months after surgery, see Figure 2. Before surgery, average CVC score was 33 points (range: 0–77). Three months after surgery, 11 cases had no improvement of CVC score (i.e. CVC‐score shift between −25 and 0), while remaining 99 cases had increased CVC scores compared with preoperative scores (range: 2–86). Cases with no residual hearing had largely same distribution of CVC‐score shift as the whole cohort. The preoperative CVC‐word test scores were comparable between groups. CVC‐score shifts were not different between HP groups at 3 and 12 months after implantation (*p *> 0.05).

## 4 DISCUSSION

This retrospective cohort study provides a complete overview of a general adult CI population of the last 5 years. Almost half of the adult patients (46%) who underwent primary cochlear implantation had residual hearing at lower frequencies. Complete HP was achieved in 10% of these patients, partial HP in 26%, minimal HP in 30% and complete loss of residual hearing was seen in 34%. LW arrays in general, and intraoperative local corticosteroids usage in small sample set of 8 patients, were associated with better HP. Speech perception of patients with preserved residual hearing was not better than patients without residual hearing after surgery. Lastly, surgical experience had no effect on HP outcomes.

### 4.1 Hearing preservation

Several different classifications are used to indicate HP at lower frequencies after CI surgery.^6^ Studies, similar to our study, described complete HP rates ranging between 0% and 68%.^7^, ^8^, ^9^ Based on these studies, and others, residual hearing at lower frequencies deteriorates over time. Direct comparison between our study and other studies is therefore somewhat limited, as most of the previously mentioned studies^8^, ^9^ measured at an earlier timepoint than our study (around 40 days vs. 88 days in this study). It is likely that HP depends on direct acute trauma during cochlear implantation resulting in inflammatory ototoxic processes, which impacts inner ear homeostasis and manifests as hearing deterioration at longer term. The deterioration over time could also be independent from cochlear implantation and might be related to progress of the disease itself. All in all, it is very difficult to establish final HP outcomes, if at all possible, considering that residual hearing is probably continuously deteriorating to some degree.

### 4.2 Patient independent factors

In our study, patients with LW arrays had more often complete HP than patients with PM arrays (14% vs. 6%). Scalar translocation is regarded as severe insertion trauma, occurring more often with PM arrays, and negatively influences residual hearing of CI recipients.^10^ Therefore, this difference is probably linked to scalar translocation. It is unknown whether these differences between LW and PM arrays remain the same on the longer term. Another factor, hyaluronic acid, had no effect on HP in our study. Another study showed a correlation between HP and the use of hyaluronic acid.^11^ However, this was a weak correlation, and is the only study, to our knowledge, showing a direct effect of hyaluronic acid on HP rates.

### 4.3 Speech perception

Preserved residual hearing can improve speech perception in patients with EAS.^2^ In our cohort, only one individual made use of EAS. We therefore looked at effect of HP on speech perception with only electrical hearing. Data regarding this relationship are, to our knowledge, scarce. We did not see an association between HP and the speech perception test without background noise. Importantly, potential benefits of preserved residual hearing could arise if speech perception with background noise was tested. We hypothesise that it is likely that trauma and inflammation caused by cochlear implantation can affect outer and inner hair cells (i.e. loss of residual hearing), and not directly the auditory nerve at the short term. The potential benefit of preserved residual hearing in the lower frequencies on speech perception, especially in difficult listening situations such as musical melody recognition and background noise, and on speech perception related factors (e.g. intonation and listening effort), remains unclear.

## 5 CONCLUSION

Approximately half of all adult CI recipients had residual hearing at lower frequencies before surgery. The majority of these patients lost their residual hearing after cochlear implantation. In current medical practice, only electrode choice seems to have a clear effect on hearing preservation rates. Much improvement is needed to preserve the residual hearing of CI recipients in the future.

## **AUTHOR** **CONTRIBUTIONS**

SJ and HT designed the work. ED and SJ acquired and analysed data. All the authors drafted, revised, approved the manuscript and agreed to be accountable for all aspects of the work.
